# Phytochemical Characterization of *Taxus baccata* L. Aril with Emphasis on Evaluation of the Antiproliferative and Pro-Apoptotic Activity of Rhodoxanthin

**DOI:** 10.3390/antiox11061039

**Published:** 2022-05-24

**Authors:** Daria-Antonia Dumitraş, Andrea Bunea, Dan Cristian Vodnar, Daniela Hanganu, Emoke Pall, Mihai Cenariu, Adrian Florin Gal, Sanda Andrei

**Affiliations:** 1 Department of Preclinical Sciences, Faculty of Veterinary Medicine, University of Agricultural Sciences and Veterinary Medicine Cluj-Napoca, 400372 Cluj-Napoca, Romania; antonia.dumitras@usamvcluj.ro (D.-A.D.); adrian.gal@usamvcluj.ro (A.F.G.); 2 Department of Biochemistry, Faculty of Animal Science and Biotechnology, University of Agricultural Sciences and Veterinary Medicine Cluj-Napoca, 400372 Cluj-Napoca, Romania; andrea.bunea@usamvcluj.ro; 3 Department of Food Science, Faculty of Food Science and Technology, University of Agricultural Sciences and Veterinary Medicine Cluj-Napoca, 400372 Cluj-Napoca, Romania; dan.vodnar@usamvcluj.ro; 4 Department of Pharmacognosy, Faculty of Pharmacy, University of Medicine and Pharmacy “Iuliu Haţieganu”, 400372 Cluj-Napoca, Romania; dhanganu@umfcluj.ro; 5 Department of Clinical Sciences, University of Agricultural Sciences and Veterinary Medicine Cluj-Napoca, 400372 Cluj-Napoca, Romania; emoke.pall@usamvcluj.ro (E.P.); mihai.cenariu@usamvcluj.ro (M.C.)

**Keywords:** *Taxus baccata* L. arils, phenolic compounds, carotenoids, rhodoxanthin, antioxidant activity, cytotoxicity, HaCaT cells, B16F10 cells

## Abstract

*Taxus baccata* L., an evergreen tree, was known until recently due to its high concentration of toxic compounds. The purpose of the present study was to focus on the only non-poisonous part, the red arils, which have recently been described as an important source of various bioactive constituents. To establish total phenolic, flavonoid, and carotenoid content, antioxidant capacity, and cytotoxic properties, two types of extracts were obtained. The chemical profile of the ethanolic extract was evaluated using chromatographic (HPLC-DAD-ESI+) and spectral (UV-Vis) methods, and the antioxidant activity of the ethanolic extract was assessed using DPPH and FRAP assays, yielding moderate results. In the second type of extract (methanol: ethyl acetate: petroleum ether (1:1:1, *v*/*v*/*v*)) we identified three carotenoids using open column chromatography and RP–PAD–HPLC, with rhodoxanthin being the most abundant. Considering the above and mainly because of the lack of information in the literature about this pigment and its biological effects, we decided to further investigate the cytotoxic activity of rhodoxanthin, the main carotenoid presented in aril, and its protective effect against H_2_O_2_-induced oxidative stress using two cell lines: human HaCaT keratinocytes and B16F10 murine malignant melanoma. The MTT and Annexin-V Apoptosis assays showed a substantial cytotoxic potential expressed in a dose-dependent manner towards the melanoma cell line, however, no obvious cytotoxic effects on human keratinocytes were noticed.

## 1. Introduction

*Taxus baccata* L. (European yew) is a tree from the Taxaceae family, a native evergreen non-resinous gymnosperm up to 20–28 m in height, widespread in Europe and Asia [1]. The red oval-shaped fruits (arils) with a gelatinous texture represents the only part of this plant that is not poisonous. These fruits have approximately 10 mm in diameter and contain only one seed. According to previous studies, these are rich in carotenoids, especially the ones with a retro structure, but also in water-soluble antioxidants from the class of polyphenols [2,3].

*Taxus baccata* L. has become very well known for its toxic active constituents (taxine alkaloids) contained in various concentrations within the plant’s parts, except the aril, with many cases of poisoning being reported over the last years [4,5,6]. Yew intoxication mostly causes humans heart failure, with various scientists reporting cases of skin irritations and dermatitis caused by yew wood also. Animals, particularly cattle, can be also harmed by the consumption of seeds, bark, or leaves. Previous studies mentioned a lethal dose of leaves for animals: 100–250 g for sheep, 500 g for cattle, 100–200 g for horses and pigs, and 30 g for dogs. Cattle, predominantly horses, are said to be sensitive, with eating yew twigs or bark proving to be fatal in the majority of cases [1]. This has resulted in the extinction of this plant, which is now difficult to find in natural environments. Unfortunately, as more knowledge about the use of this plant as a suicide technique has become available through the media, yew poisoning has become more accessible [7]. However, recent studies discovered the valuable properties of multiple active substances extracted from *Taxus* for treating several diseases, including cancer. Although it is considered a toxic plant, containing in its shoots many cardiotoxic substances, Taxol (generic name Paclitaxel) [8,9,10], which was isolated for the first time from the bark of *Taxus brevifolia Peattie* (Pacific yew), is one of the most valuable bioactive compounds due to its antitumoral properties observed especially related to ovarian and breast cancer. The extract of *Taxus baccata*’s leaves was proved to possess significant anti-asthmatic activity [11] being also mentioned in Ayurvedic Medicine as an adjuvant in asthmatic bronchitis, indigestion, malaria, and rheumatism treatment [12,13]. A high demand for Taxus was reported lately for the preparation of Paclitaxel because due to over-harvesting of its bark and leaves, most wild populations are threatened with extinction [14]. In addition, new advancements in the composition of arils have piqued scientists’ interest in the area. Their high content of important micro- and macro-elements, good quality proteins, and low content of simple carbohydrates makes it represent a valuable element of the food diet [15]. Carotenoids, particularly those with a retro structure such as rhodoxanthin, but also lycopene and zeaxanthin, have been discovered to be one of these fruits’ most important components. Rhodoxanthin is a rarely found pigment in nature, being previously reported in some fish and birds’ feathers [16]. The pigment came from the birds’ diet, the probable exogenous sources being represented by berries of two exotic Asian bush honeysuckle species, *Lonicera marrowii* and *Lonicera tatarica*. Some authors also mentioned the arils of *Taxus baccata* as part of the diet in case of some birds [1,15,17,18].

Carotenoids are lipophilic pigments and there are more than 700 types of carotenoids found in nature [19]. Carotenoids have evolved recently from just simple molecules that interact with radical species to biomarkers linked to positive outcomes in a variety of degenerative diseases, including lung, gastrointestinal tract, pancreas, breast, and prostate cancers, cardiovascular disease, and age-related macular degeneration [20,21]. Following the recent considerable advancements in knowledge regarding the production of carotenoid metabolites, the absorption and transport processes responsible for the accumulation of carotenoids in certain organs and tissues, and the expanding number of metabolic pathways in which carotenoids can function, the research of the molecular mechanisms that allow carotenoids to be involved in such multifactorial activities was most encouraged.

In the light of the recent epidemiologic studies that have reported that certain plant-based extracts containing carotenoids can be helpful by averting the development or slowing down the progress of numerous diseases, we consider that studying more the chemical composition of arils and mostly the antiproliferative activity of the major carotenoid, rhodoxanthin, which to the best of our knowledge, has not been studied in this direction, could be a step ahead for science and medicine. Considering this, we investigated the cytotoxic activity and the protective effect against H_2_O_2_-induced oxidative stress on two different cell lines: human HaCaT keratinocytes and B16F10 murine malignant melanoma.

## 2. Materials and Methods

### 2.1. Plant Samples and Growing Conditions

**Figure 1:**
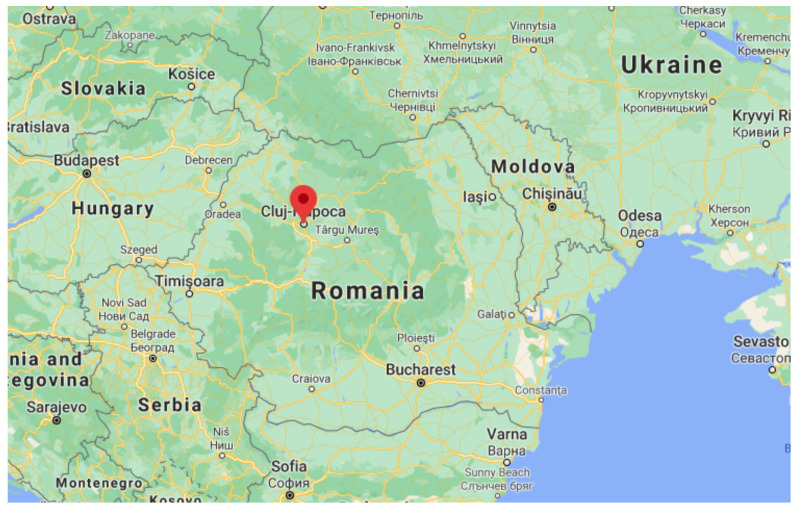
Red aryl of *Taxus baccata* L.—habitat.

Red berries (arils) of *Taxus baccata* L. were collected from plants growing in natural habitats, in the autumn (September–October) of 2020 from three different locations in Cluj-Napoca (46°46′0.01″ N, 23°36′0.00″ E), Cluj County, Romania (Figure 1) [22]: (1) University of Agricultural Sciences and Veterinary Medicine campus, (2) Alexandru Borza Cluj-Napoca University Botanic Garden, and (3) ‘Simion Bărnuțiu’ Central Park. The climate in Cluj-Napoca is moderately continental, being influenced by the city’s proximity to the Apuseni Mountains as well as by urbanization. It is characterized by cold winters with often temperatures below (0 °C or 32 °F) and mild warm summers [23,24]. The climate in September is relatively dry with 17.6 mm of rainfall, humidity of 76%, and involves a peak temperature of 21 °C and a lowest of 8 °C [25]. According to a study published in 2014 by the French magazine ‘We Demain’, the city has the best air quality in the European Union [26]. The collection areas had a modest solar exposure considering the slope exposure. We utilized only the red flesh of the fruit for the intended determinations, thus once the fruits were harvested, the toxic parts (seeds) were manually removed and the whole quantity of fruits was divided into sample bags, each weighing approximately 20 g FWA (fresh weight arils) and were stored in the freezer (−80 °C) until further processing.

### 2.2. Chemicals and Reagents

Standards of phenolic and carotenoid compounds were purchased from Sigma Aldrich (Darmstadt, Germany). Before RP–PAD–HPLC analysis, all the samples were filtered through a 0.45 µm MF-Millipore™ Membrane Filter from Merck (Darmstadt, Germany). Plant Flavonoids Colorimetric Assay Kit was purchased from Elabscience Biotechnology Inc. (Houston, TX, USA) and Annexin V-FITC and propidium iodide (PI) for flow-cytometry Kit was purchased from Thermo Fisher Scientific Inc. (Waltham, MA, USA). The remaining reagents used were acquired from Sigma Aldrich (Darmstadt, Germany) and Merck (Darmstadt, Germany).

### 2.3. Determination of Carotenoid Composition

#### 2.3.1. Preparation of Extract

The pigments were extracted from fresh arils, using a mix of methanol: ethyl acetate: petroleum ether (1:1:1, *v*/*v*/*v*), by homogenizing this for 3 min at 2500 RPM using a Vortex homogenizer (Ultra-Turrax Miccra D-9 KT Digitronic, Bergheim, Germany). Repeated extractions in the dark for 4 h with continuous stirring were performed until the residue was colorless. The united extracts were filtered and then partitioned sequentially with water, diethyl ether, and saturated NaCl solution using a separation funnel. All extraction events were accomplished under subdued light in order to avoid degradation loss of the pigments. The ether phase was evaporated by rotary evaporation, at 35 °C and the obtained oleoresin residue was dissolved in a known volume of ethyl acetate for further chromatographic analysis. All extractions were done in triplicate.

#### 2.3.2. Total Carotenoid Content

In order to determine the total carotenoid concentration in the red arils, we used a UV spectrophotometric assay. The residue obtained was dissolved in 15 mL of petroleum ether and the absorption spectrum was determined in the intervals of 300–700 nm. The determination was realized by measuring the absorbance at 442 nm. In order to calculate the total content of carotenoids, we used a mathematical formula [27]. 

#### 2.3.3. Carotenoids Analysis by Open-Column Chromatography 

With the intention of identifying the carotenoids contained, the total carotenoid extract was separated in an open column of silica gel and eluted with *n*-hexane–acetone–methanol 85:15:1 (*v*/*v*/*v*). The three colored bands separated were collected individually, concentrated in a rotary evaporator (≤35 °C), and dissolved in a known volume of hexane. In order to be able to identify the pigments present in the separated fractions, the absorption spectra in the range 300–600 nm were recorded. Pigment identification was based on comparing the characteristic absorption spectra with previous literature data [18,27,28].

#### 2.3.4. Carotenoids Analysis by RP–PAD–HPLC

HPLC carotenoids separation was carried out on the total extract and the fractions previously separated on silica gel open column chromatography according to a slightly modified method [29]. The system used was the Shimadzu Prominence LC-20 AT (Shimadzu, Kyoto, Japan) controller system (Shimadzu Corporation, Kyoto, Japan) comprising of a DGU-20A3 degasser and photodiode array detector (SPD-M20A UV–Vis). Phenomenex C30 column (24 cm × 4.6 mm; particle size: 5 µm) and a dual gradient mobile phase was used. The mobile phase consisted of a mixture of solvent (A) methanol/tert-butyl methyl ether/water (81:15:4, *v*/*v*/*v*) and solvent and (B) tert-butyl methyl ether/methanol/water (90:7:3, *v*/*v*/*v*). The gradient program started with 1% B to 100% B from 0 to 16 min and continued isocratically up to 30 min. The flow rate was 0.8 mL/min. All chromatograms were monitored at 450 nm.

### 2.4. Total and Individual Content of Phenolic Compounds

#### 2.4.1. Preparation of Extract

Each aril sample (10 g) was independently extracted three times, using 70 mL of ethanol: water (70% *v*/*v*) on a magnetic stirrer for 2 h in extremely low light exposure. Following this step, the extract was filtered and the extraction was repeated three times. In the end, the final extracts were combined and centrifuged (3500 rpm for 10 min), the supernatant was collected and the subsequent determinations were performed.

#### 2.4.2. Total Phenolic Content

The total phenolic content was determined through the Folin–Ciocalteu method [30]. For the quantification, a calibration curve of gallic acid was prepared with solutions in the range of 50–450 µg/mL (R^2^ = 0.9994). The absorbance was determined at 765 nm [31]. The results were expressed as mg of GAE (gallic acid equivalent) per 100 g FWF. The analyses were run in triplicate and carried out using the microplate spectrophotometer SPECTROstar^®^ Nano (BMG Labtech, Ortenberg, Baden-Württemberg, Germany).

#### 2.4.3. Total Flavonoid Content

Total flavonoid content (TFC) was determined using a Plant Flavonoids Colorimetric Assay Kit and the detection principle is based on the reaction between the flavonoids and the aluminum ion resulting in a red complex. The flavonoid content of the sample can be then calculated by recording the absorptivity of the sample at 510 nm. First of all, we had to prepare a standard curve following the given instructions in the kit, by diluting the 1 mg/mL standard solution with double distilled water to a serial concentration (150, 120, 100, 80, 60, and 20 µg/mL). After that, the next step consisted of preparing the sample and reading its absorbance against double-distilled water as a blank. The final result was obtained using a mathematical formula given in the kit. The results were expressed in mg QE/100 g FWF, run in triplicate, and carried out using the microplate spectrophotometer SPECTROstar^®^ Nano (BMG Labtech). 

#### 2.4.4. HPLC-DAD-ESI-MS Analysis

Identification and quantification of phenolic compounds in the red arils were performed on an HPLC-DAD-ESI-MS system involving an Agilent 1200 HPLC equipped with a DAD detector, coupled with a single quadrupole MS (Agilent 6110) detector. Separations were performed on the Eclipse column, XDB C18 (4.6 × 150 mm, particle size 5 μm) at 25 °C, using the A and B mobile phases for 30 min with a flow rate of 0.5 mL/min. The A mobile phase included 0.1% acetic acid in distilled water (*v*/*v*), while the B mobile phase contained 0.1% acetic acid in acetonitrile (*v*/*v*). The gradient program began with 5% B (0–2 min), and the percentage of mobile phase B was gradually increased as follows: 5−40% B (2–18 min), 40−90% B (18–20 min), and 90% B (30 min). After 24 min, the percentage of mobile phase B began to decrease from 90% to 5% in one minute, followed by column equilibration for 5 min [32]. The absorbance spectrum was continuously determined in the 200–600 nm interval and the phenolic acids were detected at 280 nm, while the flavonoids were at 340 nm. For the MS fragmentation it was used ESI source in the (+) mode. The capillary voltage was 3000 V, the temperature was 35 °C, with a nitrogen flow of 7 L/min. The scan range was between 100–1200 *m*/*z* and the eluent was monitored by DAD. Data analysis was carried out by Agilent ChemStation software (Chelmsford, MA, USA). 

### 2.5. Antioxidant Activity

#### 2.5.1. Determination of FRAP Scavenging Activity

The FRAP method is based on the visible color alteration of a complex with the Fe^+3^ ion of the 2,4,6-tri(2-pyridyl)-1,3,5-triazine (TPTZ) radical produced by the reduction of the ferric ion in this complex to the ferrous ion (Fe^+2^). Briefly, 2.5 mL of 10 mM TPTZ solution in 40 mM HCl was combined with 2.5 mL of 20 mM ferric chloride solution and 25 mL of acetate buffer (pH = 3.6). This blend represents the FRAP reagent. To 4.0 mL of alcoholic extract, 0.8 mL of water, and 6.0 mL of FRAP reagent were added, and similarly, a blank solution was prepared using water instead of the sample. Trolox was utilized as a reference. The color alteration was interrelated with the antioxidant activity by measuring the absorbance at 450 nm. Using a calibration curve (R^2^ = 0.992), the result was transformed to µM Trolox equivalents/100 mL extract [33].

#### 2.5.2. Determination of DPPH Scavenging Activity

The spectrophotometric method detailed by Ebrahimabadi and colab. Ref. [34] was performed (with a few modifications) to determine the DPPH free-radical–scavenging activity. Briefly, 2 mL (at different concentrations) of the ethanolic extract was added to 2 mL DPPH• (0.1 mM in 40% ethanol). After 30 min in a thermostatic bath at 40 °C, the variation of the absorbance was measured at 517 nm (R^2^ = 0.997). The DPPH radical scavenging activity of the extract was expressed as IC_50_ (mg/mL) following the monitorization of the DPPH decrease [35]. The percentage inhibition (I%) was determined according to the formula: I% = [(AB − AA)/AB] × 100, where AB = blank absorbance, and AA = aril extract absorbance.

### 2.6. Cell Cultures and Cytotoxic Assay

#### 2.6.1. Cell Lines and Cultures

B16F10 metastatic murine melanoma cell lines and human HaCaT keratinocytes were obtained from the Institute of Oncology “Prof. Dr. Ion Chiricuta” Cluj-Napoca, Romania. The cells were maintained for five days in Dulbecco’s Modified Eagle Medium supplemented with 10% fetal bovine serum, 100 IU/mL penicillin in a humidified incubator containing 5% CO_2_ incubator (Advantage-Lab, Schilde, Belgium) at 37 °C in a humidified atmosphere.

#### 2.6.2. Cell Viability Assay

Cytotoxicity study on B16F10 metastatic murine melanoma cell lines and human HaCaT keratinocytes was performed using the MTT assay (3-(4,5-dimethylthiazol-2-yl)-2,5-diphenyl tetrazolium bromide). The cells were plated (1 × 10^5^ cells/well) in 96-well plates for 24 h in normal propagation media (200 µL cell suspension in each well). Extracts were added to the complete medium in five distinct volumes (25 µL, 20 µL, 15 µL, 10 µL, and 5 µL), with the resulting concentrations (C1, C2, C3, C4, and C5) calculated according to the separated rhodoxanthin concentration determined as follows: C1—0.18 µmol/mL rhodoxanthin, C2—0.13 µmol/mL rhodoxanthin, C3—0.077 µmol/mL rhodoxanthin, C4—0.05 µmol/mL rhodoxanthin, and C5—0.025 µmol/mL rhodoxanthin. Each of the concentrations represents the rhodoxanthin quantity added in one well. The negative control was represented by cells cultured in a normal expansion medium (untreated cells). The cell viability subsequent 24 h incubation at 37 °C in a humidified atmosphere with 5% CO_2_ was assessed using the MTT assay according to previously published protocols [36,37]. The formazan particles formed by adding 0.5 mg MTT to each well were dissolved with 200 µL dimethyl sulfoxide (DMSO), and the absorbance was determined at 450 nm using a microplate reader (Bio-Rad, Hercules, CA, USA). The cell viability percentages (%) were calculated based on the absorbance ratio between cell cultures treated with extracts and the negative controls (untreated cells) multiplied by 100. All experiments were performed in triplicates.

In order to assess the protective effect of rhodoxanthin on H_2_O_2_-induced cytotoxicity, both cell types were initially treated with rhodoxanthin as previously described, but in this case, we only used the first three concentrations (C1—0.18 µmol/mL rhodoxanthin, C2—0.13 µmol/mL rhodoxanthin, and C3—0.077 µmol/mL rhodoxanthin) due to their stronger effect formerly determined. After 24 h, the medium was discarded and subsequently cells were treated with three different concentrations of hydrogen peroxide (200 µM, 500 µM, and 1000 µM H_2_O_2_ for 2 h). In addition, both cell types were exposed to the three concentrations of hydrogen peroxide for 2 h and cell viability was determined. The percentage of viable cells was calculated following the formula previously mentioned.

#### 2.6.3. Apoptosis Assay

A dead cell apoptosis kit with Annexin V-FITC and propidium iodide (PI), for flow-cytometry was used in order to assess the cytotoxic activity of rhodoxanthin. A number of 2.5 × 10^5^ cells/well were seeded in 12-well plates and treated with the same concentrations of rhodoxanthin mentioned before (C1–C5) for HaCaT keratinocytes cells and B16F10 murine melanoma cells, respectively. C1–C3 were used for the cells that were previously exposed to H_2_O_2_. The dead cell apoptosis kit was used in accordance with the manufacturer’s instructions. After 24 h of incubation, the cells were trypsinized, and the cell suspensions were rinsed with cold PBS and resuspended in 100 L binding buffer. 5 L Annexin V-FITC and 1 L PI (100 g/mL propidium iodide working solution) were added to each sample. Samples were incubated in low-light conditions for 15 min before being examined with a flow cytometer equipped with a 488 nm, blue, air-cooled, 20 mW solid-state excitation laser. Fluorescence was detected using the 530/30 FITC filter and the 575/26 PI filter. The FACSDiva 6.1.2 software (Becton Dickinson, San Jose, CA, USA) was used for the analysis. It was considered that Annexin V-FITC-positive alone signals an early stage of apoptosis, whereas double staining (Annexin V-FITC/PI) suggests a late, more advanced stage, of apoptosis. Single PI-stained cells were deemed necrotic, whereas unstained cells were deemed viable.

### 2.7. Statistical Analysis

All measurements were performed in triplicate and the results were expressed as mean ± SD. Statistical analyses were completed with the GraphPad Prism 8 statistics program (GraphPad Software, San Diego, CA, USA). Data statistical analyses were achieved by using one-way ANOVA. The level of significance was set at *p* < 0.05.

## 3. Results and Discussions

### 3.1. Phytochemicals Content in Taxus baccata L. Aril

*Taxus baccata* arils were previously reported as important sources of various bioactive substances [38] and high quality macro- and micro-nutrients [9]. Hence, investigating the content and various biological effects of these phytonutrients is of paramount importance. In our study, we focused on identifying and quantifying the bioactive compounds that can have a potential antioxidant and antiproliferative activity.

Table 1 summarises our information regarding the total carotenoid content, the major carotenoid identified, the rhodoxanthin content, the total phenolic compounds content, and the total flavonoid content.

#### 3.1.1. Carotenoids Content

**Figure 2:**
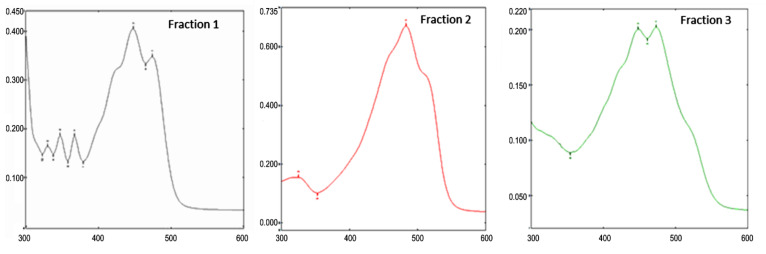
Absorption spectra of chromatographically separated fractions from aril extracts of *Taxus baccata*.

Separation of the major carotenoid pigments and obtaining the rhodoxanthin fractions were performed by silica gel column chromatography. By elution, the presence of three different fractions was observed: fraction 1—narrow, yellow band; fraction 2—the major one, intensely colored in red; and fraction 3—narrow, orange band. In order to identify the pigments in the separate fractions, the absorption spectra in the range of 300–600 nm were recorded (Figure 2), and the data were correlated with those obtained in HPLC analysis.

**Figure 3:**
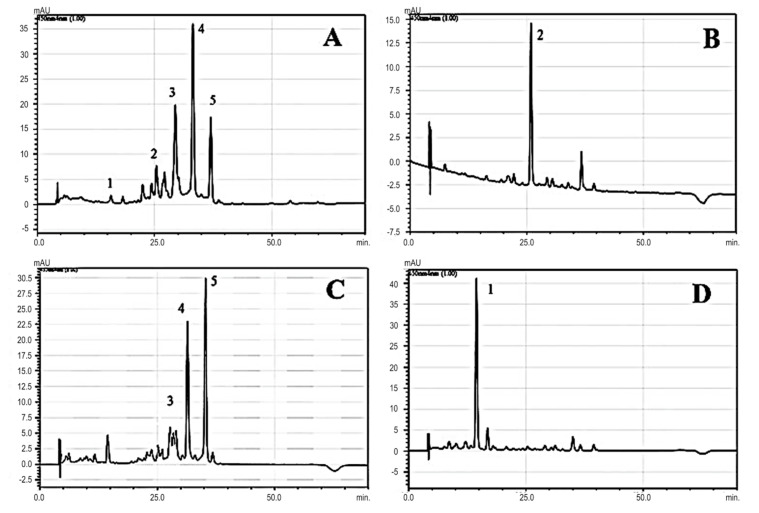
HPLC chromatogram of carotenoids in the total extract obtained by fresh arils of *Taxus baccata* (**A**) and fractions (fraction 1—(**B**); fraction 2—(**C**); and fraction 3—(**D**)). Five carotenoids are identified: (1) lutein, (2) β-carotene, (3), and (4) and (5) rhodoxanthin isomers.

The HPLC chromatograms for the carotenoids separated from the fresh aril of *Taxus baccata* total extract (A) and the fractions separated on open column (B; C; D) are presented in Figure 3. Five compounds were identified in the *Taxus baccata* arils: lutein, β-carotene, and three rhodoxanthin isomers (Table 2 and Figure 3). The HPLC peaks were identified either by using parallel HPLC runs with carotenoid standards (lutein and beta-carotene), as well as by recording the UV–Vis spectra specific to each carotenoid peak and their comparison with known carotenoid spectra and previous studies [18,27,28].

According to the literature, rhodoxanthin is a carotenoid with the property of high isomerization, upon dissolution in solvents and mild thermal treatments at the double bond 6 (6′) of the ionic cycle, forming a mixture of three different geometric isomers: the all-trans isomer, 6-mono-cis, and 6, 6′-di-cis [27,28].

According to a recent study [28], *Taxus* aril contains a number of 21 carotenoids (in different isomeric forms) the most important being the retro-structured carotenoids, rhodoxanthin, and eschscholtzxanthin, and the less abundant violaxanthin, zeaxanthin, β-cryptoxanthin, lutein, and β-carotene. The data presented also show that the major pigment is rhodoxanthin, which displayed a main absorption maxima (λmax) ranging from 478 to 502 nm across the rhodoxanthin (E/Z)-isomers assessed, being detected at 502 nm for (all-E)-rhodoxanthin as well as at 494 and 486 nm for (6Z)- and (6Z,6′Z)-rhodoxanthin, respectively. 

Following the identification of the major pigments, a semi-quantitative analysis was performed by integrating the separate peaks. The analysis was completed on the total extract to determine the total percentage of rhodoxanthin and also for fraction 2 in order to determine the purity of the rhodoxanthin fraction separated. In the *Taxus baccata* L. fresh aril total extract, the major carotenoids were represented by the rhodoxanthin isomers, summing about 77% of the total carotenoid content. In the case of fraction 2, the values obtained after integration showed that the purity of rhodoxanthin isomers was high, the total sum of the isomers of rhodoxanthin representing 89.70%.

#### 3.1.2. Total Phenolic and Total Flavonoid Contents

The total polyphenols content was 145.71 ± 22.3648 mg GAE/100 g FWF as mentioned in the Table 1.

HPLC-PDA-MS identification of phenolic compounds was made based on their retention time, UV–VIS spectra, and mass spectral analysis compared with standards and literature data. Results obtained for the HPLC-DAD-ESI+ analysis of total phenolic compounds can be found in Table 3. The determination of the polyphenol profile revealed the presence of 6 compounds belonging to 3 different classes as follows: p-coumarin acid, protocatechuic acid, hydroxy-caffeic acid, catechin-glucoside, and another compound that has not been identified. To calculate the concentration of the identified phenolic compounds, a calibration curve was performed with 99% purity gallic acid (Sigma); Y = 40.7 × X − 71.5 (R^2^ = 0.9995).

Among the identified compounds, protocatechuic acid, which belongs to the class of hydroxybenzoic acids, had the highest concentration (10.528 μg/mL extract). Close to its value is catechin glucoside, a compound that belongs to the subclass of flavanols, with a concentration of 9.51 μg/mL extract.

### 3.2. Antioxidant Activity

The in vitro antioxidant capacity of the aril’s ethanolic extract containing phenolic compounds, was evaluated by two different methods: the 2,2-diphenyl-picrylhydrazil (DPPH•) scavenging assay and ferric-reducing antioxidant power (FRAP). Our choice in methods relied on their different mechanisms to prove the antioxidant capacity. Due to the lack of information on the antioxidant activity of bioactive compounds in aril, we decided to compare our results to those of various fruits already known for their content of phytonutrients and valuable antioxidant activity.

Antioxidant activity of the ethanolic extract using the DPPH bleaching assay, revealed a moderate antioxidant activity with IC_50_ = 68.46 mg/mL. Regarding the data in the literature related to the antioxidant activity of compounds extracted from other fruits, the values obtained by researchers for blackcurrant, redcurrant, and gooseberry are lower, respectively, but close to the value obtained in our study [39]. The result of the FRAP test is expressed in μM TE/100 mL extract. The value obtained by us for aril (10.7 μmol/100 mL) is close to the value reported [40] for elderberry fruits (10 μmol/100 mL).

The present study offers originality and the first information regarding the antioxidant capacity of the tested samples from aril. The antioxidant capacity of the tested samples is highly related to their phenolic composition [41,42] as it is widely known and accepted that phenylpropanoids, flavonoids, and secoiridoids have antioxidant and antiproliferative properties. 

Due to the moderate antioxidant capacity exerted by the phenolic compounds in the aril, we decided to pursue the possible cytotoxic effect of the other main class of bioactive substances in the aril, the carotenoids, known for their effectiveness against multiple cancer cell lines [43], focusing on the main one in the aril, rhodoxanthin. 

### 3.3. Cell Cultures and Cytotoxic Assay

Our choice to focus our study on investigating the effects of rhodoxanthin on two cell lines, human HaCaT keratinocytes and B16F10 murine malignant melanoma cells, is based on two important reasons. The first is, as mentioned earlier, the low amount of polyphenols contained in aril, and the second is the advantage that carotenoids have in chemoprevention [44], not to mention the fact that rhodoxanthin, the carotenoid of our major interest, from our knowledge so far, has not been studied in this direction.

#### 3.3.1. Cytotoxic Activity of Rhodoxanthin on B16F10 Metastatic Murine Melanoma and Human HaCaT Keratinocytes 

**Figure 4:**
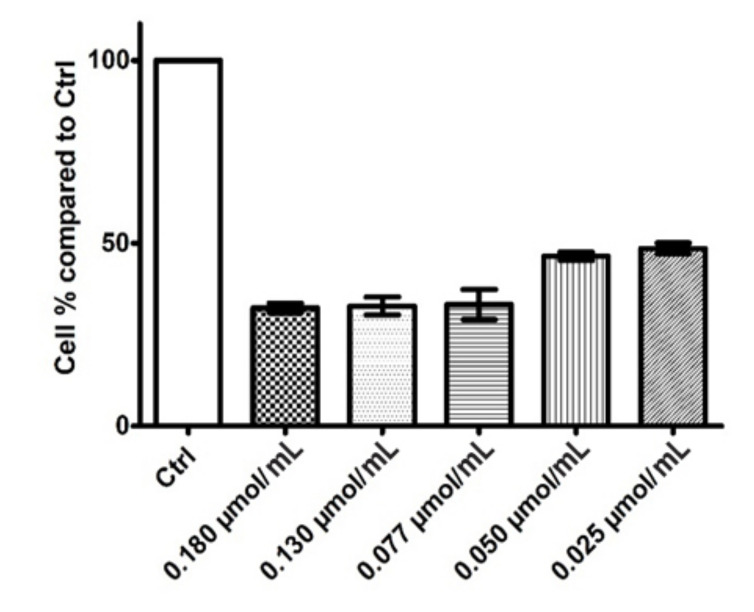
Inhibitory effect of rhodoxanthin at five different concentrations (C1–C5) on B16F10 murine melanoma cells. Ctrl—untreated cells. Values represent the mean ± SD of three determinations. *p* < 0.001.

**Figure 5:**
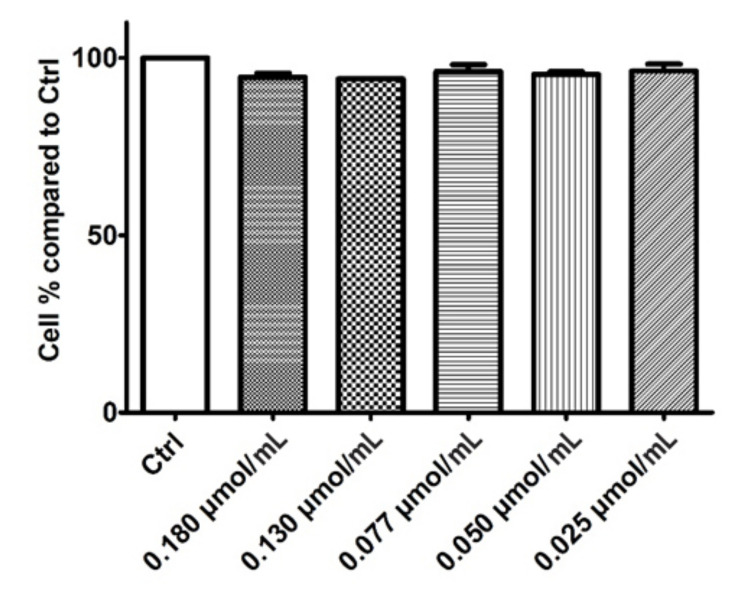
Inhibitory effect of rhodoxanthin at five different concentrations (C1–C5) on HaCaT cells. Ctrl—untreated cells. Values represent the mean ± SD of three determinations. *p* > 0.05.

To fulfill the goal of investigating the in vitro antiproliferative potential of rhodoxanthin extract from red aril of *Taxus baccata*, MTT assay and PI Annexin-V. MTT results for the five rhodoxanthin concentrations (C1–C5) tested on both cell lines are presented in Figure 4 and Figure 5. The performed determination revealed a relevant in vitro cytotoxic activity for all the doses tested on the murine melanoma cell line, the cell viability being statistically (*p* < 0.0001) diminished in a dose-dependent manner, hence the highest concentration tested (C1 = 0.18 µmol/mL) lead to 32.29% cell viability, whereas the lowest concentration of rhodoxanthin, (C5 = 0.025 µmol/mL) led to a drop in cell viability up to 48.58%. On the other hand, none of the five doses of rhodoxanthin had obvious cytotoxic effects on human keratinocytes, the viability remaining close to the untreated ones, hence ranging from 94.55% (C1 = 0.18 µmol/mL rhodoxanthin) to 96.28% (C5 = 0.025 µmol/mL rhodoxanthin).

**Figure 6:**
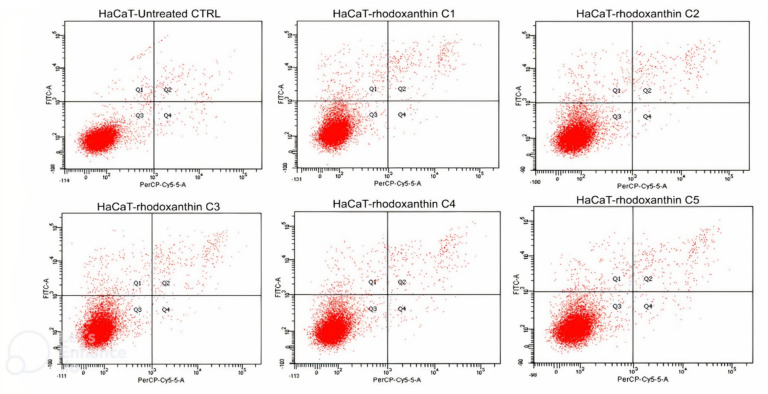
Analysis by flow cytometry of the viability of the HaCaT cell line treated with rhodoxanthin (Q1 = cells in early apoptosis, Q2 = cells in late apoptosis, Q3 = viable cells, and Q4 = necrotic cells). Values represent the mean ± SD of three samples (C1—0.18 µmol/mL, C2—0.13 µmol/mL, C3—0.077 µmol/mL, C4—0.05 µmol/mL, and C5—0.025 µmol/mL rhodoxanthin).

**Figure 7:**
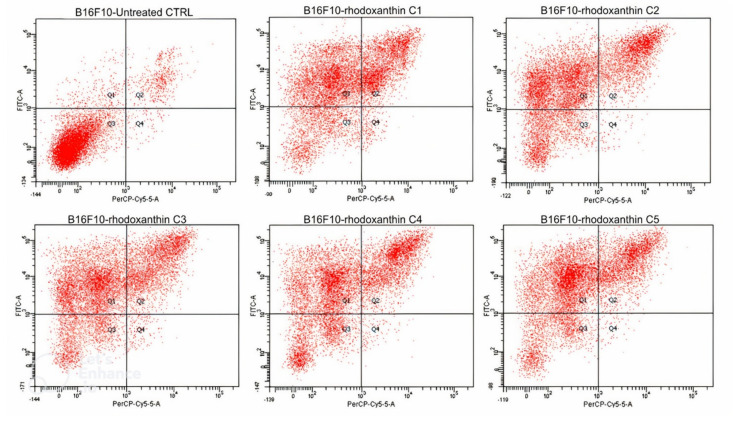
Analysis by flow cytometry of the viability of the B16F10 cell line treated with rhodoxanthin (Q1 = cells in early apoptosis, Q2 = cells in late apoptosis, Q3 = viable cells, and Q4 = necrotic cells). Values represent the mean ± SD of three samples (C1—0.18 µmol/mL, C2—0.13 µmol/mL, C3—0.077 µmol/mL, C4—0.05 µmol/mL, and C5—0.025 µmol/mL rhodoxanthin).

**Figure 8:**
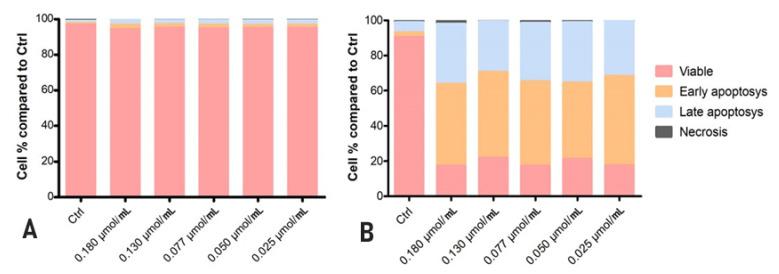
(**A**) Flow-cytometry analysis of HaCaT cells following exposure to rhodoxanthin. (**B**) Flow-cytometry analysis of B16F10 cells following exposure to rhodoxanthin. Values represent the mean ± SD of three samples.

The previously described results were further confirmed by flow cytometry followed by a PE Annexin-V cell stain. In the HaCaT cells case, we demonstrated that rhodoxanthin does not significantly affect the viability of keratinocytes, the evolution of the cells being similar to the control group. Thus, in the control group (untreated cells), 97.2% of the cells in the sample were viable, while cells treated with the highest concentration of rhodoxanthin (C1 = 0.18 µmol/mL rhodoxanthin), 95.3% remained viable after treatment, only 1.8% of the cells were in early apoptosis, 2.4% of the cells were in late apoptosis, and 1.8% were represented by necrotic cells. As expected, the results were different for B16F10 melanoma cells. Therefore, we observed significant changes in the viability of rhodoxanthin-treated cells in all concentrations used. If the control group represented 90.4% viable cells, this percentage decreased in a dose-dependent manner. In group 1 (C5 = 0.025 µmol/mL rhodoxanthin), only 17.4% of the cells maintained their viability, while 46.4% were in early apoptosis, 34.2% were in late apoptosis, and 2.1% of the cells were necrotic. Following treatment with C4 (0.05 µmol/mL rhodoxanthin), 21.7% of the cells were viable, 48.8% were in early apoptosis, 28.8% in late apoptosis, and the remaining 0.6% were necrotic cells. The results obtained for the sample of cells treated with C3 (0.077 µmol/mL rhodoxanthin) were as follows: 17.3% of the cells were viable, 47.9% of the cells were in the stage of early apoptosis, 33.2% were in the stage of late apoptosis, and 1.6% were necrotic. The second concentration of rhodoxanthin tested (C2 = 0.13 µmol/mL rhodoxanthin) resulted in 21.1% viable cells, 43.3% in early apoptosis, and 34.5% in delayed apoptosis. Following exposure to C5, we observed the highest percentage of cells in the stage of early apoptosis, namely 50.8%, while only 17.4% of the cells remained viable. Thus, it is without a doubt that *Taxus baccata* aril extract has a strong cytotoxic effect on murine melanoma B16F10 cells, while in the case of human keratinocytes, cytotoxicity is lower. The details are presented in Figure 6, Figure 7 and Figure 8.

#### 3.3.2. Cytoprotective Effects of Rhodoxanthin against Hydrogen Peroxide-Induced Oxidative Stress in Human Keratinocytes 

One of the most common types of reactive oxygen species (ROS), hydrogen peroxide, has been used in experimental models to induce oxidative stress and cytotoxicity. Previous research indicates that dietary hydrolysable tannins, such as ellagitannins and gallotannins can reduce H_2_O_2_-induced cell death in human skin cells [19,45].

**Figure 9:**
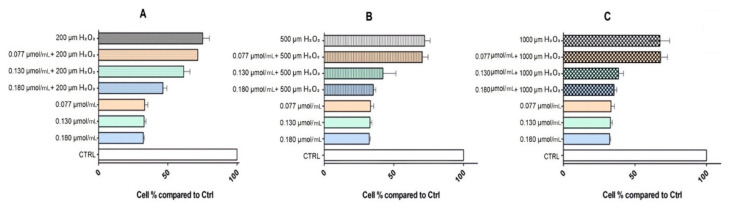
Effects of rhodoxanthin pretreatment on H_2_O_2_-induced B16F10 cells damage. Cell viability detected by MTT assay. B16F10 cells exposed to H_2_O_2_ for 2 h. Significance: *p* < 0.0001 vs. control. B16F10 cells exposed to C1–C3 of rhodoxanthin for 24 h. Significance: *p* < 0.0010. (**A**) B16F10 cells exposed to C1–C3 and 200 µm H_2_O_2_. Significance: *p* < 0.05. (**B**) B16f10 cells exposed to C1–C3 and 500 µm H_2_O_2_. Significance: *p* < 0.05. (**C**) B16F10 cells exposed to C1–C3 and 1000 µm H_2_O_2_. Significance: *p* < 0.005.

**Figure 10:**
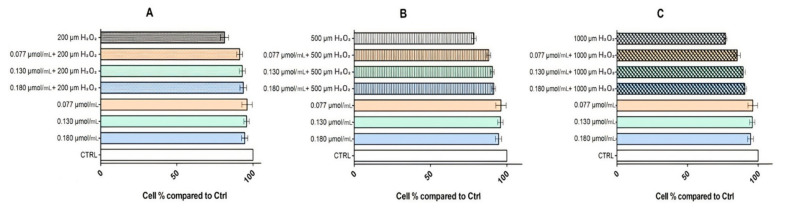
Effects of rhodoxanthin pretreatment on H_2_O_2_-induced HaCaT cells damage. Cell viability detected by MTT assay. HaCaT cells exposed to H_2_O_2_ for 2 h. Significance: *p* < 0.0001 vs. control. HaCaT cells exposed to C1–C3 of rhodoxanthin for 24 h. Significance: *p* < 0.0010. (**A**) HaCaT cells exposed to C1–C3 and 200 µm H_2_O_2_. Significance: *p* < 0.05. (**B**) HaCaT cells exposed to C1–C3 and 500 µm H_2_O_2_. Significance: *p* < 0.05. (**C**) HaCaT cells exposed to C1–C3 and 1000 µm H_2_O_2_. Significance: *p* < 0.005.

An MTT test was also performed in order to explore the oxidative protection potential of rhodoxanthin on the same cell lines, testing the first three concentrations previously mentioned (C1 = 0.18 µmol/mL rhodoxanthin, C2 = 0.13 µmol/mL rhodoxanthin, and C3 = 0.077 µmol/mL rhodoxanthin). Briefly, pretreatment with rhodoxanthin at nominal concentrations (0.18 µmol/mL, 0.13 µmol/mL, and 0.077 µmol/mL) for 24 h, of the cells subsequently exposed to three concentrations (200 µm, 500 µm, and 1000 µm) of H_2_O_2_ resulted in the data shown in Figure 9 and Figure 10. Hydrogen peroxide exposure induced cytotoxicity in HaCaT cells by reducing the cell viability up to 81.30% (200 µM H_2_O_2_), 78.41% (500 µM H_2_O_2_), and 76.89% (1000 µM H_2_O_2_), respectively. Treatment with rhodoxanthin showed a cytoprotective effect on HaCaT cells exposed to H_2_O_2_, with all three concentrations of rhodoxanthin (C1–C3) increasing the cell viability by 12.55%, 13%, and 9.66%, respectively. 

The three concentrations of hydrogen peroxide to which B16F10 melanoma cells were exposed led to a decrease in their viability as follows: 75.23% at 200 µM H_2_O_2_ exposure, 72.04% at 500 µM H_2_O_2_ exposure, and 67.41% at an exposure of 1000 µM H_2_O_2_. On the other hand, the action of the three rhodoxanthin concentrations tested on B16F10 cells led to the following viability percentages: C1—32.29%, C2—32.82%, and C3—33.26%. As a result of the pretreatment with rhodoxanthin and the exposure to 200 µM H_2_O_2_ of melanoma cells, a synergistic action of the two compounds was observed, an action that generated a decrease in cell viability as follows: C1 + 200 µM H_2_O_2_—46.45%, C2 + 200 µM H_2_O_2_—61.51%, and C3 + 200 µM H_2_O_2_—33.26% cells viable. The combination of pre-treatment with rhodoxanthin and exposure to 500 µM H_2_O_2_ led to the following cell viability values: C1 + 500 µM H_2_O_2_—35.21% viable cells, C2 + 500 µM H_2_O_2_—42.10%, and C3 + 500 µM H_2_O_2_—70.43%, and in the case of the highest concentration of hydrogen peroxide used, 1000 µM H_2_O_2_, combined with rhodoxanthin pretreatment, cell viability decreased to: C1 + 1000 µM H_2_O_2_—35.43% viable cells, C2 + 1000 µM H_2_O_2_—38.47%, and C3 + 1000 µM H_2_O_2_—68.87%.

Previous studies [46] mention the protective effect of rhodoxanthin against H_2_O_2_-induced oxidative stress in human retinal epithelial (RPE) cells. While H_2_O_2_ exposure led to a decrease in cell viability up to 73.22%, following treatment with rhodoxanthin, the cell viability increased to 97.81%.

**Figure 11:**
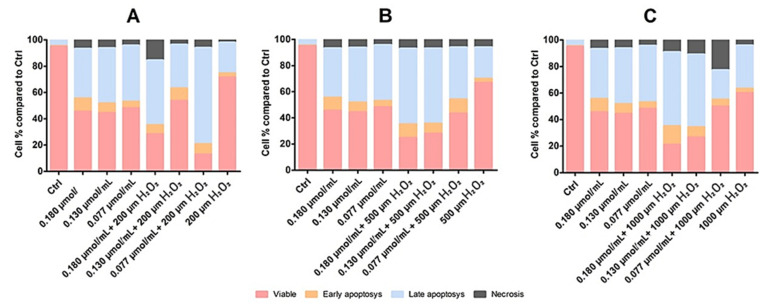
Flow-cytometry analysis of B16F10 cells following rhodoxanthin and hydrogen peroxide exposure. (**A**) B16F10 cells exposed to C1–C3 and 200 µm H_2_O_2_. Significance: *p* < 0.05. (**B**) B16f10 cells exposed to C1–C3 and 500 µm H_2_O_2_. Significance: *p* < 0.05. (**C**) B16F10 cells exposed to C1–C3 and 1000 µm H_2_O_2_. Significance: *p* < 0.005. Values represent the mean ± SD of three samples.

Flow cytometry analysis also revealed a synergistic action of rhodoxanthin and H_2_O_2_ against B16F10 melanoma cells, by effectively reducing the viable cell percentage. Rhodoxanthin showed cytotoxic effects by reducing even more cell viability; the higher the concentration of rhodoxanthin, the lower the viability of melanoma cells. The rate of viable cells in the B16F10 control group was 94.9%, which was reduced after exposure to H_2_O_2_ as follows: 200 µM H_2_O_2_—71.5%, 500 µM H_2_O_2_—66.8%, and 1000 µM H_2_O_2_—60.1%. (Figure 11).

Hydrogen peroxide was previously reported as a valuable molecule that could be helpful in the treatment of cancer. Recently [47], a group of scientists investigated the effect of hydrogen peroxide 33% topical solution (HP33) as a neo-adjuvant in a study conducted on 12 subjects diagnosed with non-melanoma skin cancer undergoing surgical excision. They reported a statistically significant reduction in the size of a variety of skin cancers, including squamous and basal cell carcinomas, after a single application of HP33. Hydrogen peroxide was also described as a selective killer of the cancer cells while being harmless to the normal cells, due to their mechanisms of defense. Hence, the synergy was demonstrated between a natural enzyme-like product that converts superoxide dismutase to hydrogen peroxide and radiotherapy in the treatment of various tumors [48].

Another study proved the implication of hydrogen peroxide in inhibiting cell growth. As a result, green tea catechins (−)-epigallocatechin-3-gallate (EGCG) and (−)-epigallocatechin (EGC) exhibited powerful growth inhibitory effects against the lung tumor cell lines H661 and H1299 by means of being hydrogen peroxide-induced, which showed to mediate cell apoptosis [49]. However, to our knowledge, the synergistic action between a retro-carotenoid and hydrogen peroxide in inducing the apoptosis of some tumor cells has not been previously demonstrated.

## 4. Conclusions

To the best of our knowledge, this is the first study aimed at assessing the chemical profile, antioxidant capacity, and chemopreventive properties of multiple types of extracts obtained from the arils of *Taxus baccata* L. 

The biochemical profile of *Taxus baccata* arils revealed the presence of phenolic compounds in trace amounts, in a total quantity of 145.71 ± 22.3648 mg GAE/100 g FWF. Following the two methods used to assess antioxidant capacity, FRAP and DPPH, the phenolic compounds extracted from the arils were found to exert a moderate antioxidant activity (IC_50_ = 68.46 mg/mL). Carotenoids, on the other hand, accounted for the majority of the bioactive chemicals isolated from arils. The presence of three pigments, beta-carotene, lutein, and rhodoxanthin, was identified, with rhodoxanthin being the most abundant, accounting for 77% of the overall carotenoid content. Based on the results of the carotenoid profile analysis and the fact that there are several studies in the literature on the multiple biological effects of beta-carotene and lutein, we considered it important to conduct a more detailed study of rhodoxanthin, a lesser-known pigment, in terms of antiproliferative, antioxidant, and pro-apoptotic capacity.

As a result, the effect of five rhodoxanthin concentrations was evaluated on two cell lines: human HaCaT keratinocytes and B16F10 murine melanoma cells. Rhodoxanthin exhibited considerable cytotoxicity in B16F10 murine melanoma cells at all dosages tested, with cell viability ranging between 48.58% and 32.29%. Rhodoxanthin had almost no cytotoxic effect on HaCaT cell viability, which ranged from 94.55% to 96.28%. MTT and Annexin-V Apoptosis assay tests were also performed on the same cell lines in order to investigate the oxidative protection potential of rhodoxanthin pretreatment, revealing a protective effect against H_2_O_2_-induced oxidative stress in human HaCaT keratinocytes, increasing the cell viability by 12.55%, 13%, and 9.66%, respectively, compared to the cell groups exposed to the three concentrations of H_2_O_2_ used (200 µm—81.30%, 500 µm—78.41%, and 1000 µm—76.89%). A synergistic action of rhodoxanthin and H_2_O_2_ against B16F10 melanoma cells by effectively reducing the viable cell percentage was observed, hence, rhodoxanthin showed a cytotoxic effect by reducing even more the murine melanoma cell viability; the higher the concentration of rhodoxanthin, the lower the viability of melanoma cells.

Without wishing to diminish the importance of this study, further studies are required to clarify the mechanisms of action behind the cytotoxic effect of rhodoxanthin in cancer cells, but we consider the obtained results very valuable for pointing out in a new direction of research.

## Figures and Tables

**Table 1:** Phytochemicals Content in *Taxus baccata* Aril.

Analyzed Samples	Total Carotenoids mg/100 g FW	Rhodoxanthin mg/100 g FW	Total Polyphenols mg GAE/100 g FWF	Flavonoids mg QE/100 g FWF
**Sample 1**	3.384	2.536	146.69	43.95
**Sample 2**	3.371	2.582	146.64	45.01
**Sample 3**	3.379	2.575	143.8	45.26
**Average ± SD**	**3.378 ± 0.0053**	**2.564 ± 1.0079**	**145.71 ± 22.3648**	**44.723 ± 15.4256**

**Table 2:** HPLC peaks and corresponding compounds identified in red arils.

Compound	Peak	RT (min)	Max Absorption (nm)
Lutein	1	14.44	421,445,474
Beta-Carotene	2	25.41	425,450,477
Rhodoxanthin isomer	3	29.45	337,487
Rhodoxanthin isomer	4	33.21	337,487
Rhodoxanthin isomer	5	37.01	337,503

**Table 3:** Quantification and identification of phenolic compounds (µg/g FWA) by HPLC-DAD-ESI+ analysis.

Peak No.	Compound	Retention Time R_t_ (min)	UV λ_max_ (nm)	[M + H]^+^ (*m*/*z*)	Subclass	Concentration (μg/g)
1	NI	3.30	230	381, *219*		42.2
2	*p*-Coumaric acid-glucoside	6.12	320	327, *166*	Hydroxycinnamic acid	1046.235
3	Protocatechuic acid	9.62	290	155	Hydroxybenzoic acid	149.49
4	Hydroxy-caffeic acid	12.20	322	197	Hydroxycinnamic acid	49.37
5	Caffeic acid	13.78	322	181	Hydroxycinnamic acid	95.76
6	Catechin-glucoside	16.45	280	453, *291*	Flavanol	131.36

The mean values are obtained from triplicate determinations and expressed on a fresh weight (FW) basis (NI = not identified).

## Data Availability

The data presented in this study are available in the article.
